# New Artificial Diet for Continuous Rearing of the Bean Pod Borer, *Maruca vitrata*

**DOI:** 10.1673/031.013.12101

**Published:** 2013-11-05

**Authors:** Pan Wang, Peng-Fei Lu, Xia-Lin Zheng, Li-Zhen Chen, Chao-Liang Lei, Xiao-Ping Wang

**Affiliations:** 1 Hubei Insect Resources Utilization and Sustainable Pest Management Key Laboratory, College of Plant Science and Technology, Huazhong Agricultural University, Wuhan 430070, P. R. China; 2 The Key Laboratory for Silviculture and Conservation of Ministry of Education, Beijing Forestry University, Beijing 100083, P. R. China

**Keywords:** reproductive potential

## Abstract

The bean pod borer, *Maruca vitrata* Fabricius (Lepidoptera: Crambidae), is a serious pantropical pest of grain legumes. A suitable artificial diet is desirable for producing uniform insects for commercial purposes or research. Three previously described artificial diets, 1 newly-developed artificial diet, and cowpea (*Vigna unguiculata* (L.) Walp. (Fabales: Fabaceae)), the natural hostplant of *M. vitrata*, were used for rearing *M. vitrata*, and the life parameters were examined. The results indicated that insects completed a full life cycle only when the larvae were fed cowpea or the diet reported by Onyango and Ochieng'-Odero (1993), called the “D-OO diet.” However, the rearing efficiency (i.e., larval and pupal survival, longevity of adults, and fecundity) on the D-OO diet was inferior to the rearing efficiency on cowpea. Subsequently, a new artificial diet was formulated based on soybean powder, *Glycine max* (L.) Merr. (Fabales: Fabaceae), and wheat germ, *Triticum aestivum* L. (Poales: Poaceae). The egg production, egg hatching, larval developmental duration, and pupal survival of the *M. vitrata* reared on the new artificial diet were found to be significantly improved relative to the D-OO diet, but were not significantly better than on the host-plant cowpea. The optimum rearing density was 15–25 larvae per box. There were no significant changes in reproductive potential after 8 successive generations of rearing on the new diet. These results indicated that the newly developed diet could serve as a viable alternative to cowpea plant for continuous rearing of *M. vitrata*.

## Introduction

The bean pod borer, *Maruca vitrata* Fabricius (Lepidoptera: Crambidae), is an important pantropical pest of grain legumes, and has a wide distribution throughout Africa, Asia, South America, and the southern states of Australia (Sharma 1998). In China, *M. vitrata* is the primary boring pest of leguminous vegetable crops, particularly the cowpea, *Vigna unguiculata* (L.) Walpers (Fabales: Fabaceae), and has caused significant yield losses yearly (Ke et al. 1985). Chemical insecticides are widely used to control this pest (Afun et al. 1991; Asante et al. 2001). However, the cryptic behavior of the larvae, the cost, and the environmental risks associated with chemical insecticides impose serious limitations on the use of the chemicals to control this species (Singh and Allen 1980; Jackai and Daoust 1986). Effective control of *M. vitrata* often relies on sound integrated pest management strategies. In order to develop and optimize strategies, studies must be focused on the biology, bionomics, behaviors, and ecology of *M. vitrata*, and on improving cowpea resistance against *M. vitrata*. One priority to conduct these studies is the availability of a continuous and adequate supply of high quality experimental insects.

Many studies have focused on developing an economical diet formula and cost-effective rearing techniques for *M. vitrata* (Jackai and Raulston 1982, 1988; Onyango and Ochieng'- Odero 1993; Chi et al. 2004; Liu and Hwang 2006; Jin et al. 2010). However, there are many questions and issues related to the development of artificial diets for the continuous rearing of this species. First, some of the components are difficult to find or obtain, such as cowpea flower powder (Onyango and Ochieng'-Odero 1993), Insecta F-II (Chi et al. 2004), Taiwan sesbania leaves, and Taiwan sesbania seed powder (Liu and Hwang 2006). Second, the rearing density of the larvae in most previous studies was 1 larvae per container (Jackai and Raulston 1988; Chi et al. 2004) or less than 5 larvae per container (Onyango and Ochieng'-Odero 1993; Jin et al. 2010), except for Liu and Hwang (2006). Third, it is difficult to obtain an adequate number of mating pairs to maintain the experimental population by using an artificial diet (Jackai and Raulston 1988). Therefore, artificial diets for this species need to be improved for continuous rearing in the laboratory to produce a large amount of uniform insects.

The aim of this study was to develop an artificial diet suitable for the continuous rearing of *M. vitrata* without a loss of vigor or reproductive potential.

## Materials and Methods

### Experimental insects

*M. vitrata* larvae were collected from the Cihui farm (30° 59′ N, 114° 06′ E) in Wuhan City, Hubei Province, P. R. China. The population was maintained on the host-plant cowpea at 26 ± 1° C, with a photoperiod of 14:10 L:D and 60 ± 10% RH, except for adult mating and oviposition, when they were kept at at 85–90% RH. The eggs were collected daily, and the newly hatched larvae were used in the following experiments.

Twenty newly hatched larvae were transferred to transparent plastic boxes (10 cm diameter) and reared with either artificial diets or on cowpea. The artificial diet was replaced every 5–6 days, and the cowpea was replaced every 2 days. A corrugated paper was put into the box as a pupation site when the larvae entered the prepupal stage, which was determined by a change in the body color from pink to light green. The pupae were sexed and kept indi vidually in plastic boxes. For mating, 15 pairs of newly emerged moths were transferred into a rearing cage (length × width × height = 30 × 40 × 50 cm) with a 10% honey solution. After 3 days, each pair was kept separately in an ovipositing container with a 10% honey solution. The container was composed of a transparent cylindrical plastic cup (8cm diameter, 9 cm height) with a Petri dish base on the bottom. Oviposition occurred on the inner surface of the cup. The cups were replaced daily. The cups with eggs were kept at 26 ± 1° C, with a photoperiod of 14:10 L:D and 60 ± 10% RH until hatching.

### Diet ingredients source

The ingredients of all the diets are shown in Table 1. Soybean, *Glycine max* (L.) Merrill (Fabales: Fabaceae), and wheat germ, *Triticum aestivum Triticum aestivum* L. (Poales: Poaceae), were purchased from a local market. Wesson salt mix and vitamin mixture were prepared as the detailed list shown in the footnote of Table 1. Brewers' yeast was purchased from Angel Yeast Corporation Ltd. (www.angelyeast.com). Other ingredients (industrial grade products) were ordered from China National Medicine Corporation, Ltd. (www.cncm.com.cn).

### Experiment 1: Re-evaluation of the reported Diets

Three diets described in previous studies (Table 1) were selected to determine their suitability for continuous rearing. The availability of their components and their reported rearing efficiency were also assessed. These artificial diets were prepared following the procedures described in the previous studies (Jackai and Raulston 1988 (D-JR); Onyango and Ochieng'-Odero 1993 (D-OO); Jin et al. 2010 (D-J)). Life parameters were measured, including the survival rates, the developmental durations of the larvae and pupae, longevity, egg production, egg hatching, and egg developmental duration. Twenty larvae were reared in 1 container, as described by Liu and Hwang (2006). Each diet was replicated 10 times. Eight pairs of adult for each diet were used to measure the adult longevity and egg production.

### Experiment 2: Evaluation of the new artificial Diet

**Figure 1.**
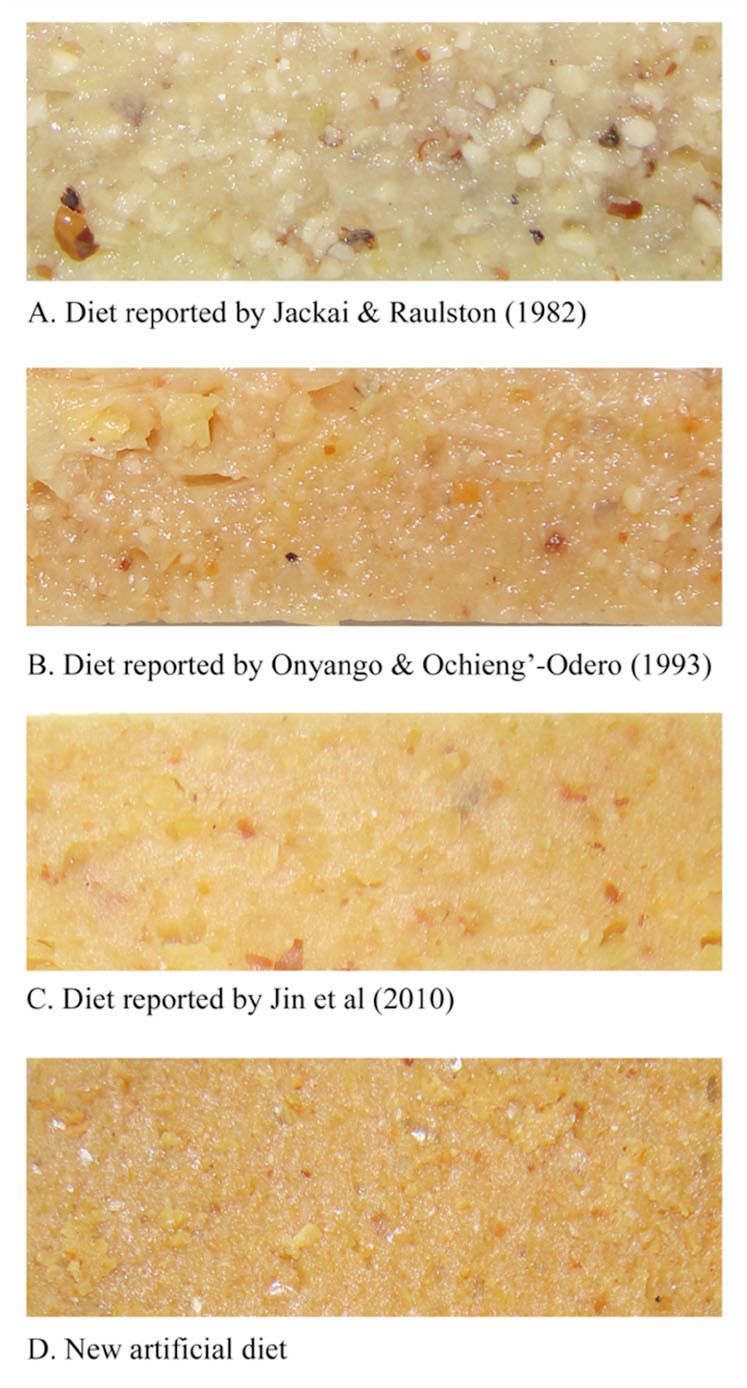
Three reported artificial diets and a new artificial diet for continuous rearing of *Maruca vitrata*. High quality figures are available online.

The results of Experiment 1 showed that enough adults for continuous rearing were obtained only when the larvae were fed on the D-OO diet. However, compared to the hostplant cowpea, the pupal survival, egg production, and egg hatching were lower and the developmental duration of the immature stage was longer on the D-OO diet. The diet was improved by changing the nutritional composition, shown in Table 1. Expensive components, such as cowpea flower powder, cowpea leaf factor, and cowpea flour were eliminated. A new artificial diet (D-New) was obtained after a series of preliminary experiments (Table 1, Figure 1D).

**Table 1.**
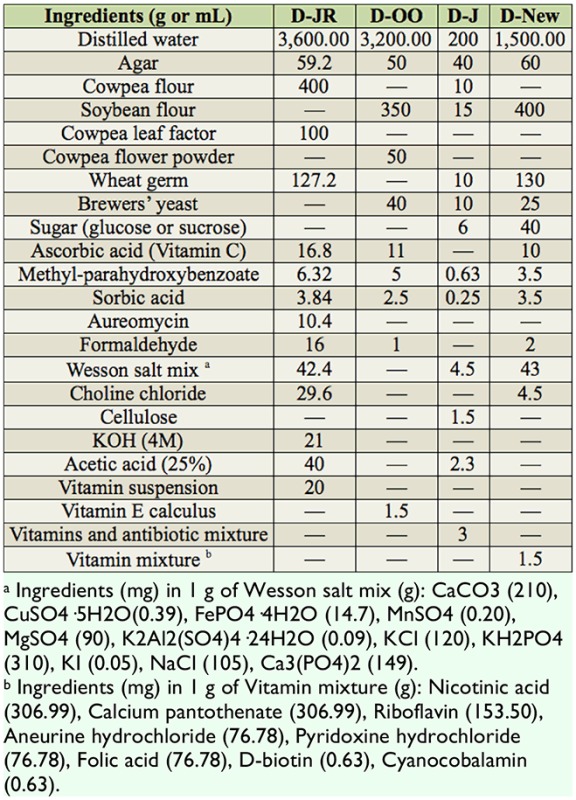
Formula of 3 reported artificial diets and a new artificial diet for continuous rearing of *Maruca vitrata*.

### Preparation of the new artificial diet

The new artificial diet was prepared in 5 steps as follows: (1) soybeans were baked in an oven at 100° C for 6 hr and ground to a fine powder in a 250 mL stainless steel blender; (2) agar was combined with 1/4 of the final volume of water; (3) the major nutritional and antimicrobial ingredients, soybean flour, wheat germ, sucrose, brewer's yeast, sorbic acid, and methyl-parahydroxybenzoate, were combined and added to the remaining volume of the water; (4) the first 2 parts (2 and 3) of the diet were blended and stirred for 3 min separately, then autoclaved at 121° C and 15 psi for 15 min together. After autoclaving, the 2 parts were blended for 5–6 min in the blender until cooled to approximately 60° C; then, the third part of the diet, including Wesson salt mix, ascorbic acid, choline chloride, a vitamin mixture, and approximately 50 mL of distilled water, was added to the mixture. Finally, (5) formaldehyde was added to the ingredients and blended for 2 min. After mixing all the ingredients and cooling to room temperature, the paste-like diet was dispensed into appropriate containers and stored at 4° C.

### Comparison of the efficiency of continuous Rearing

The suitability of D-New for continuous rearing was estimated by comparing its effectiveness with those reared on cowpea. Life parameters were measured in terms of survival rates, developmental durations of the larvae and pupae, longevity, egg production, egg hatching, and egg developmental duration. Twenty larvae were reared in 1 box, as described by Liu and Hwang (2006). Each diet was replicated 10 times. Eight pairs of adults for each diet were used to measure the adult longevity and egg production.

### Effect of the larval rearing density

To evaluate the effect of larval rearing density on the survival rate, developmental duration, and pupal weight of *M. vitrata*, 5 densities (10, 15, 20, 25, and 30 larvae per box, 10 cm diameter) were used to determine the optimum larval density for continuous rearing. Insect rearing methods were the same as described above, and 150 larvae were used in each density.

### Rearing successive generations on the diet

To evaluate the effect of D-New on the reproductive potential of *M. vitrata*, all of the life parameters were compared for 8 successive generations on D-New. The methods of estimation were the same as described above, and the larval rearing density was 20 larvae per box. Ten boxes were reared for each generation.

**Table 2.**
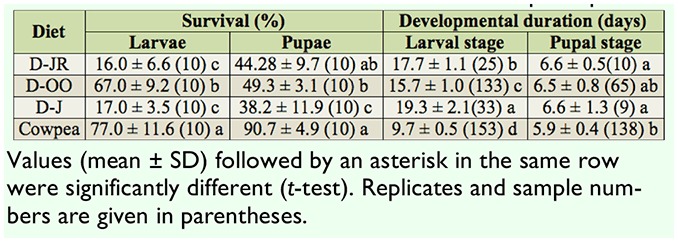
Survival and developmental duration of *Maruca vitrata* reared on different diets at 26° C with a 14:10 L:D photoperiod.

### Statistical analysis

Statistical analyses were performed using SPSS 11.5 (SPSS Inc., www-01.ibm.com/software/analytics/spss). The data for larvae and pupae survival, egg hatching, developmental duration, body weight, longevity, and fecundity were analyzed by one-way analysis of variance (ANOVA), and Tukey's HSD test (*p* < 0.05) was used to compare the means. The data for all the life parameters measured on the artificial diet and the cowpea were analyzed using an independent-samples *t* test. Prior to the analysis, an arcsine squareroot transformation was performed on the data related to the egg hatching and survival of the larvae and pupae.

**Figure 2.**
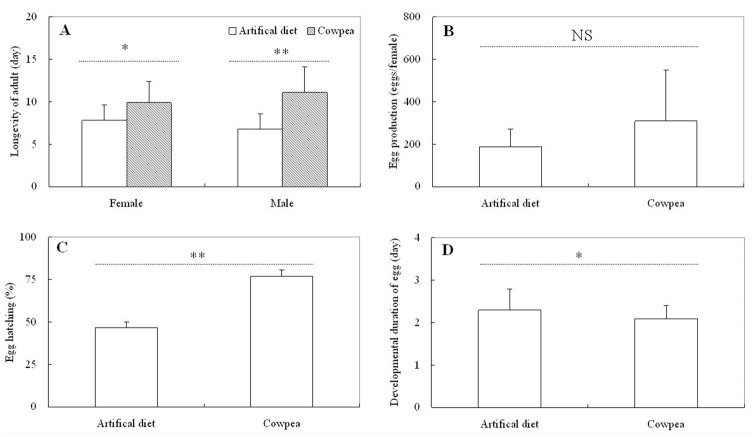
Adult longevity (A), egg production (B), egg hatching (C), and developmental duration of the eggs (D) of *Maruca vitrata* reared on the D-OO diet and on the host-plant cowpea at 26° C with a 14:10 L:D photoperiod. The error bars indicate the SD. *, *p* < 0.05; **, *p* < 0.01; NS, not significant (*t*-test). High quality figures are available online.

## Results

### Experiment 1: Re-evaluation of the reported Diets

The data on the survival and developmental duration of *M. vitrata* reared on 3 reported diets and on cowpea are given in Table 2. The survival rates of *M. vitrata* reared on 3 diets were significantly lower than those on cowpea (larvae: *F* = 152.101; df = 3, 39; *p* = 0.000; pupae: *F* = 84.746; df = 3, 39; *p* = 0.000). The developmental duration of *M. vitrata* reared on 3 diets was significantly longer than those on cowpea (larvae: *F* = 1439.538; df = 3, 343; *p* = 0.000; pupae: *F* = 13.288; df = 3, 221; *p* = 0.000). For all the parameters measured in this experiment, the results of D-OO were superior to those D-JR and D-J, even though the differences were not statistically significant in some cases (e.g., for pupal developmental duration). Furthermore, enough adults for continuous rearing were obtained only for the larvae fed on D-OO or reared on cowpea. Figure 2 displays the adult longevity (Figure 2A), egg production (Figure 2B), egg hatching (Figure 2C), and developmental duration of the eggs (Figure 2D) of *M. vitrata* reared on the D-OO diet and on host-plant cowpea. Except for egg production, the measures of the three other parameters measured for the D-OO diet were not higher than the measures for the same parameters associated with rearing on the hostplant cowpea (Figure 2).

**Table 3.**
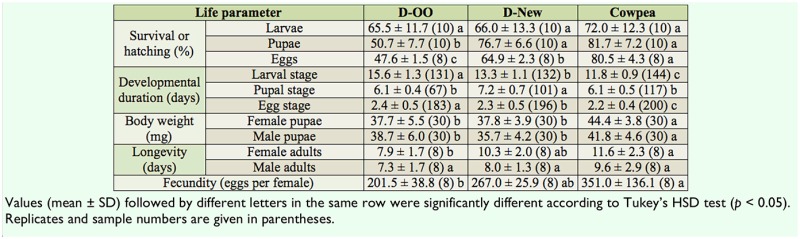
Comparison of life parameters of *Maruca vitrata* reared on the D-OO, D-New, and cowpea diets at 26° C with a 14:10 L:D photoperiod.

### Experiment 2: Evaluation of the new artificial Diet

#### Comparison of the efficiency of continuous Rearing

The comparisons of all the life parameters of *M. vitrata* reared on D-OO, D-New, and hostplant cowpea are shown in Table 3. Larval survival was not significantly different for *M. vitrata* reared on these three diets. Pupal survival and egg hatching for insects reared on D-New were significantly higher than for those on D-OO, but were lower than those on host-plant cowpea (pupal survival: *F* = 50.054; df = 2, 29; *p* = 0.000; egg hatching: *F* = 255.768; df = 2, 23; *p* = 0.000). The developmental durations of eggs and larvae on DNew were significantly shorter than those reared on D-OO but significantly longer than those reared on host-plant cowpea (eggs: *F* = 12.403; df = 2, 578; *p* = 0.000; larvae: *F* = 398.266; df = 2, 406; *p* = 0.000). The developmental duration of pupae on D-New was significantly longer than for those on the other two diets (*F* = 121.494; df = 2, 284; *p* = 0.000). Pupal body weight on host-plant cowpea was significantly greater than that on DOO and D-New (female pupae: *F* = 22.297; df = 2, 89; *p* = 0.000; male pupae: *F* = 11.242; df = 2, 89; *p* = 0.000). Male adult longevity for *M. vitrata* reared on these three diets changed slightly (*F* = 2.771; df = 2, 23; *p* = 0.086). Female adult longevity on D-New was longer than that on D-OO and shorter than that on host-plant cowpea, but the difference was significant only between D-OO and host-plant cowpea (*F* = 7.177; df = 2, 23; *p* = 0.004). The fecundity of females obtained on D-New was significantly greater than those on D-OO. Although females reared on D-New produced fewer eggs than those reared on host-plant cowpea, the difference was not statistically significant (Table 3: *F* = 6.512; df = 2, 23; *p* = 0.006).

### Effect of the larval rearing density

The effects of larval rearing density on survival, the developmental duration of the larvae and pupae, and the pupal weight are shown in Table 4. Larval rearing density had no significant effect on the survival of the larvae and the pupae (Table 4; larvae: *F* = 1.893; df = 4, 43; *p* = 0.131; pupae: *F* = 0.325; df = 4, 43; *p* = 0.860). Pupal developmental times for the *M. vitrata* reared in medium density (15–25 larvae per box) were shorter than for those reared in high density (30 larvae per box) and low density (10 larvae per box) (Table 4; *F* = 4.200; df = 4, 329; *p* = 0.002), whereas such differences were absent at the larval stage (Table 4; *F* = 2.508, df = 4, 458; *p* = 0.041). There were no significant differences among the body weights of male pupae reared at different densities (Table 4; *F* = 1.518; df = 4, 129; *p* = 0.201), whereas the body weights of the female pupae decreased with an increase in larval density from 10 to 30 larvae per box (Table 4; *F* = 2.632; df = 4, 129; *p* = 0.037).

**Table 4.**
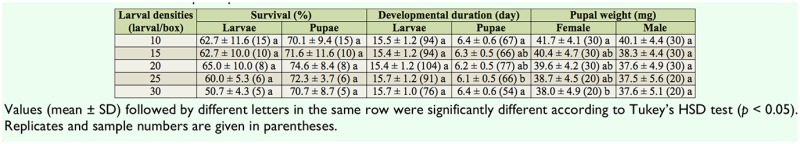
Effect of the larval rearing density on the survival, developmental duration, and pupal weight of *Maruca vitrata* reared on DNew at 26° C with a 14:10 L:D photoperiod.

**Table 5.**
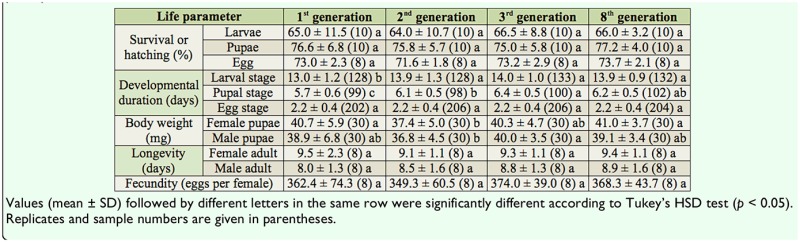
Life parameters of *Maruca vitrata* reared on the new artificial diet in succession for 8 generations at 26° C with a 14:10 L:D photoperiod.

### Rearing successive generations on the new artificial diet

The biological performances of the 8 successive generations fed D-New were compared by measuring survival and hatching, developmental duration, body weight, longevity, and fecundity. Except for developmental duration and body weight, no decline in the survival rate and/or reproductive potential was found after 8 successive generations (Table 5).

## Discussion

This study demonstrated that *M. vitrata* performed excellently on D-New compared to other artificial diets, but not compared to hostplant cowpea. Some life parameters of insects reared on D-New, including immature survival, adult longevity, fecundity, and egg hatchability, were similar to those on hostplant cowpea, but the other parameters studied were significantly worse. These results imply that the new artificial diet based on soybean flour, wheat germ, brewers' yeast, and sucrose can be used as an alternative to natural hosts for rearing purposes.

Many factors in the rearing process could affect the performance of *M. vitrata* on artificial diet, e.g., proportions of different nutritional ingredients, physical conditions of the diet (including moisture levels and irregularities of the diet surface), microbial contamination, several specific compounds, and environmental conditions in culture rooms. The main protein sources in D-OO were soybean flour and brewer's yeast. Besides soybean flour (Blanco et al. 2009) and brewer's yeast (Savopoulou-Soultani et al. 1994), wheat germ and sucrose were also important protein components of insect's artificial diets. Fat soluble vitamins and other substances found in wheat germ might also have a beneficial effect on insects (Vanderzant 1974). Sucrose was the main energy material of herbivorous insects (Wang et al. 1984), also acting as the most important feeding stimulant (Albert et al. 1982; Wang et al. 1984), particularly during early larval development (Savopoulou-Soultani et al. 1994). In addition to sucrose, ascorbic acid has been found to act as feeding stimulant (Dadd 1957; Montenegro de Sales 1972) and is a nutritional requirement (Ito 1961) for many species. The results of our study indicate that including wheat germ, sucrose, and the content of brewer's yeast and ascorbic acid can provide sufficient nutrition for normal insect growth, survival, and fecundity, and sustain completions of multiple life cycles. A high water content in a diet can cause drowning of larvae and asphyxiation of adults (Wang et al. 1984; Singh and Moore 1985). Lingappa (1987) found that low agar content in the diet increased the larval mortality. In our observation, a high mortality rate appeared in the larval stage, especially in the neonate larval stage. This result could at least partially be attributed to the inappropriate water and agar content in the D-J and D-JR.

Maintaining high-quality and contaminationfree diet over a long period is an important feature of artificial diets for insects. It is a common practice to use antimicrobial compounds, separately or combined, to prevent microbial contamination of synthetic diets for insects. The use of different antimicrobials was a breakthrough in the diet reported by Onyango and Ochieng'-Odero (1993). The addition of antimicrobial compounds in our artificial diet effectively prevented contamination by insecticidal pathogens for 8–10 days. Our study demonstrated that the bacteria were readily suppressed with the appropriate proportion of antimicrobial compounds, but that mold contamination was much more difficult to suppress, especially during the late larval development stage. *M. vitrata* could be healthily raised in the same boxes from neonate to pupae with only one diet block (4 × 2 × 0.2 cm) replacement because of the excellent preservatives, thus greatly reducing the frequency of diet changes and mechanical injury to the developing larvae.

Previous studies also showed that Wesson salt (Wang et al. 1984; Willis and Allen 1999) and choline chloride (Ichiki et al. 2009) were indispensable in insect diets, and may determine the growth and survival of insects (Wang et al. 1984). Wesson salt is a mixture of eleven different salts used in artificial diets for insects (Willis and Allen 1999), and contains all recommended minerals for insect diets (Wang et al. 1984). Choline chloride is related to reproduction, affecting egg and sperm production (Ichiki et al. 2009). Our preliminary experiments indicated that the addition of Wesson salt mix and choline chloride was helpful.

Larval rearing density influenced the larval survival (Gindin et al. 2009). Usually, larval density is positively correlated with mortality (especially for cannibalistic insects) and with longer duration of larval developmental (Agnew et al. 2002; Gibbs et al. 2004). The results from our study indicated that the *M. vitrata* reared in a medium density condition (15–25 larvae per box, 10 cm diameter) had a shorter developmental duration time from lar vae to pupae, while the survival rate did not differ significantly among the larvae raised at different densities. This study also revealed that one of the causes of larval mortality was cannibalism of both young and mature larvae, mostly during molting.

Reproductive potential (e.g., fecundity and egg hatching) is influenced by many factors, such as sex ratio (Stewart and Philogène 1983), delayed female mating (Foster and Ayers 1996; Proshold 1996), ages of the virgin female and male at mating (Michereff et al. 2004), and the number times the male has previously mated (Michereff et al. 2004). However, in our study, no decline was observed after 8 successive generations of laboratory rearing, and the fecundity of the *M. vitrata* females was higher than that found in Onyango and Ochieng'-Odero's work (1993), in which the rearing density was 5 larvae per container (7.5 × 2.5 cm). Therefore, we consider that D-New and a medium rearing density (15–25 larvae per box, 10 cm diameter) are suitable for continuous rearing. In addition, our research indicated that relative humidity in the culture chamber was very important during mating. For *M. vitrata*, the relative humidity needs to be maintained at 85–90% or else adults fail to mate.

In conclusion, this study demonstrated that the new artificial diet supported a high rate of survival comparable to those reared on the host-plant (cowpea). The new artificial diet was suitable for the continuous rearing of *M. vitrata* to produce uniform insects of predictable performance without a loss of vigor or a decline in reproductive potential.

## Abbreviations

**D-J**,: diet reported by Jin et al. (2010)
**D-JR**,: diet reported by Jackai and Raulston (1988)
**D-New**,: new artificial diet
**D-OO**,: diet reported by Onyango and Ochieng'-Odero (1993)
